# Fascin Inhibitors Decrease Cell Migration and Adhesion While Increase Overall Survival of Mice Bearing Bladder Cancers

**DOI:** 10.3390/cancers13112698

**Published:** 2021-05-30

**Authors:** Zhankui Zhao, Yufeng Wang, J. Jillian Zhang, Xin-Yun Huang

**Affiliations:** 1 Department of Physiology and Biophysics, Weill Cornell Medical College of Cornell University, New York, NY 10065, USA; zhaozhankuimd@mail.jnmc.edu.cn (Z.Z.); yufengwang.l@gmail.com (Y.W.); 2 Novita Pharmaceuticals, Inc., New York, NY 10065, USA; jjzhang@novita-pharm.com; 3 Sandra and Edward Meyer Cancer Center, Weill Cornell Medical College of Cornell University, New York, NY 10065, USA

**Keywords:** fascin, tumor metastasis, cytoskeleton, bladder cancer, cisplatin, anti-PD-1

## Abstract

**Simple Summary:**

Fascin is an actin-bundling protein, and is highly expressed in metastatic tumor cells. Small molecule fascin inhibitors have been recently developed to block tumor cell migration, invasion, and metastasis. Here we have tested a new fascin inhibitor on bladder cancer cells, and showed the inhibitory effects of the fascin inhibitor on bladder cancer cell migration, adhesion, and primary tumor growth. Therefore, fascin inhibitors might provide clinical benefits to bladder cancer patients.

**Abstract:**

Bladder cancer is one of the most common cancers in the world. Early stage bladder tumors can be surgically removed, but these patients usually have relapses. When bladder cancer becomes metastatic, survival is very low. There is an urgent need for new treatments for metastatic bladder cancers. Here, we report that a new fascin inhibitor decreases the migration and adhesion of bladder cancer cells. Furthermore, this inhibitor decreases the primary tumor growth and increases the overall survival of mice bearing bladder cancers, alone, as well as in combination with the chemotherapy medication, cisplatin, or the immune checkpoint inhibitor, anti-PD-1 antibody. These data suggest that fascin inhibitors can be explored as a new treatment for bladder cancers.

## 1. Introduction

Bladder cancer is the malignant growth of cells that make up the urinary bladder [1]. Urothelial carcinoma (also named as transitional cell carcinoma) is the most common type of bladder cancer. The urothelial cells line the inside of the bladder. About half of a million adults in the world were diagnosed with bladder cancer in 2018, and ~200,000 died from this disease [2]. Smoking accounts for almost half of all of these cases. Among men, bladder cancer is the fourth most common cancer in the USA. The 5-year survival rate of patients with bladder cancer that has not spread beyond the inner layer of the bladder wall is 96% [3]. However, if the bladder cancer has spread to distant parts of the body, the 5-year survival rate is 5% [3]. Therefore, prevention and delay of tumor metastasis will greatly increase the survival of bladder cancer patients. Bladder cancer treatment is determined by stage, and can be treated by surgery, chemotherapy (including cisplatin and fluorouracil (5-FU)), radiation therapy, immunotherapy (including anti-PD-1 antibody therapeutics), and targeted therapy (including FGFR inhibitor) [4,5]. Early stage bladder tumors can often be surgically removed. For metastatic bladder cancer, platinum-based chemotherapy (such as cisplatin) is usually the initial treatment. These treatment options have greatly advanced the cares of bladder cancer patients. However, the survival rate for metastatic bladder cancer patients is still very low. Effective treatments are few. New treatments for metastatic bladder cancers are urgently needed.

Tumor cell migration is essential for metastasis [6]. Migration enables tumor cells to leave the primary tumor bed, enter blood vessels, and then exit the circulation and infiltrate distant tissues/organs. Cell migration requires actin cytoskeleton reorganization by forming polymers and bundles which result in significant reshaping of the cell membrane [7]. Of the most dynamic changes in plasma membrane are the protrusions by filopodia, finger-like structures which are fundamental to the shape and motility of cells [8]. Metastatic tumor cells contain an abundance of filopodia, and their invasiveness correlates with the numbers of filopodia [8,9,10]. Filopodia are formed by actin bundles, and fascin is essential for cross-linking actin filaments into actin bundles in tumor cells. There is no amino acid sequence homology between fascin and other actin-binding proteins [11,12,13,14,15,16]. Studies in cancer patients showed that fascin is a biomarker of metastases and can be a good therapeutic target [17,18,19,20,21,22,23]. Many types of metastatic tumors have elevated fascin levels which are correlated with worsening clinical outcomes, such as aggressive phenotypes, poor prognosis, and shorter survival [24,25]. While fascin level is low or absent in normal adult epithelial cells, it is highly expressed in metastatic tumors [26,27]. Genetic studies in mice demonstrate normal development in fascin gene-knockout mice, which is likely due to the functional compensation of other actin-bundling proteins during embryonic development [28]. Fascin gene deletion could delay tumor development, including slowing of tumor growth and reducing metastatic colonization, as well as increasing overall survival [29]. In contrast, over-expression of fascin could increase tumor progression and decrease the overall survival in mouse models [30]. All together, these mouse genetic studies provide strong evidence for the essential roles that fascin plays in tumor initiation (tumor burden), tumor progression, tumor metastasis, and overall survival.

Through screening of chemical libraries, we had previously identified small molecule chemical compounds that could specifically inhibit the actin-bundling activity of fascin [31]. After optimization of one of the initial hits through medicinal chemistry, one fascin inhibitor showed improved activity in blocking actin-binding and actin-bundling activities of fascin, as well as inhibiting the migration, invasion, and metastasis of tumor cells [32]. To further understand the molecular mechanism of the fascin inhibitors, we solved the X-ray crystal structure of the complex containing both fascin and a fascin inhibitor, and the data showed that the fascin inhibitor can directly occupy one of the actin-binding sites in fascin, and induce a conformational change in the tertiary structure of fascin leading to the loss of the actin-bundling function of fascin [33]. Here, we extend our previous studies of fascin inhibitors in triple-negative breast cancers, and explore the applications of fascin inhibitors in bladder cancers. Similar to triple-negative breast cancers, fascin inhibitors block the migration of bladder cancer cells. Different from breast cancers, fascin inhibitors also decrease the primary tumor growth of bladder cancers in mouse models. Furthermore, we have shown here that fascin inhibitors act additively with cisplatin to increase the overall survival of mice bearing bladder cancers. Moreover, fascin inhibitors markedly increase the response rate to the anti-PD-1 antibody in syngeneic mouse models of bladder cancers. These data demonstrate that fascin inhibitors can be explored as a novel treatment for bladder cancers.

## 2. Results

### 2.1. Fascin Inhibitor Decreases the Migration of Bladder Cancer Cells

**Figure 1:**
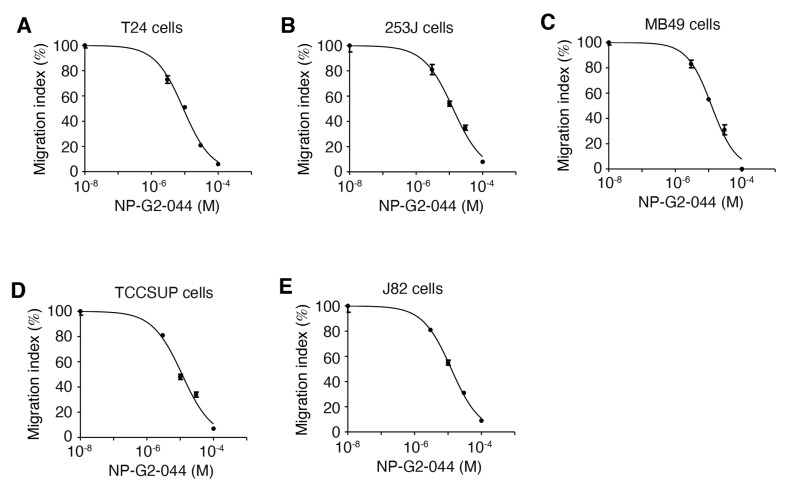
NP-G2-044 decreases the migration of bladder cancer cells. Boyden chamber migration assay was used to quantify the inhibitory effect of NP-G2-044 on the migration of various bladder cancer cells. Different concentrations of NP-G2-044 were used. Migration index was calculated using the number of migrated cells in the presence of NP-G2-044 divided by the number of migrated cells in the absence of NP-G2-044. (**A**) T24 human bladder cancer cells. (**B**) 253J human bladder cancer cells. (**C**) MB49 mouse bladder cancer cells. (**D**) TCCSUP human bladder cancer cells. (**E**) J82 human bladder cancer cells. The data were analyzed and fitted using GraphPad. Data are presented as mean ± SEM. *n* = 3.

Given the roles of fascin in actin cytoskeletal reorganization, filapodial formation, and tumor cell migration, we started with the investigation of the possible effect of fascin inhibitors on the migration of bladder carcinoma cells. To test the effect of fascin inhibitors on bladder carcinoma cell migration, we studied the migration of these cells in the absence or presence of fascin inhibitors. We used the quantitative Boyden chamber assay. Human bladder cancer cell lines were selected to represent poorly differentiated tumors (T24, 253J, and TCCSUP), and squamous differentiated tumors (J82). These cell lines have different molecular profiles [34]. T24, 253J, TCCSUP, and J82 human urinary bladder carcinoma cells, as well as MB49 mouse bladder carcinoma cells were loaded onto the top of the Boyden chamber. Cells migrated into the bottom of the chamber filter were counted. Different concentrations of the fascin inhibitor NP-G2-044 were used [32,33]. NP-G2-044 blocked the migration of all of these bladder cancer cells with IC_50_ values from 9 to 13 μM (Figure 1A–E). The actual IC_50_ values for free NP-G2-044 are 0.27–0.39 μM (in the presence of 10% of serum), given that NP-G2-044 has an ~99.7% mouse plasma protein binding [35,36]. Hence, fascin inhibitors can inhibit the migration of bladder carcinoma cells.

### 2.2. Effects of Fascin Inhibitors on the Growth of Bladder Cancer Cells

**Figure 2:**
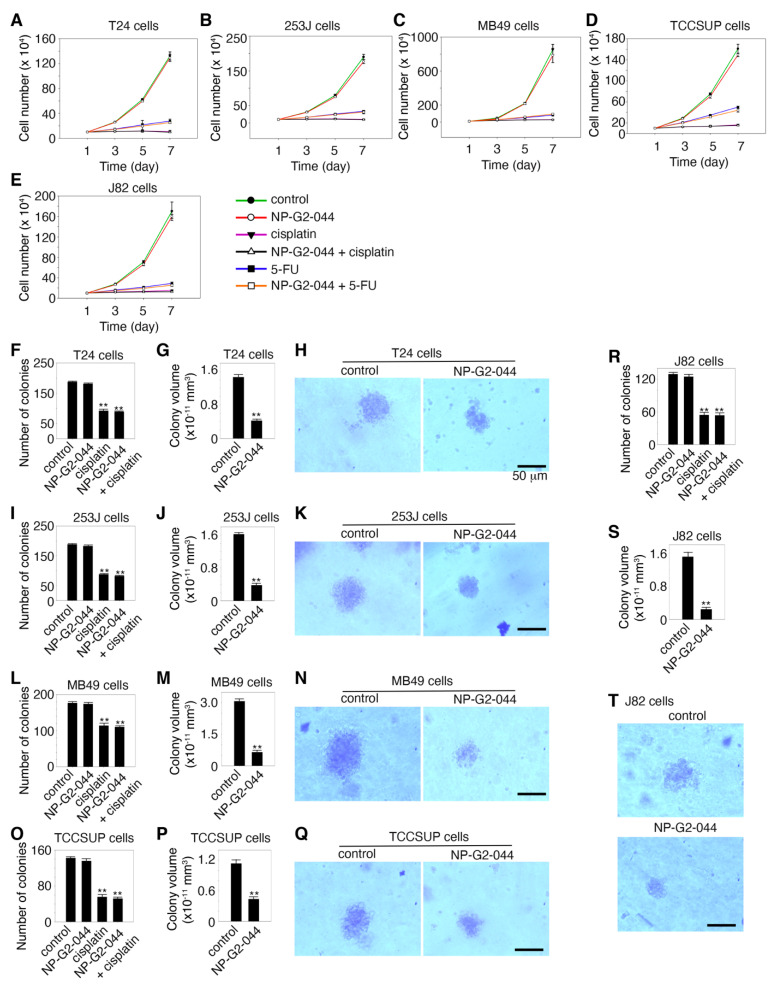
Effects of NP-G2-044 on the growth of bladder cancer cells. (**A**–**E**) Effect of NP-G2-044 on the growth of various bladder cancer cells in culture plates under 2D conditions. Cisplatin and 5-FU were used as positive control. Untreated and treated bladder tumor cells grew in the presence of 10% serum, and the number of cells was counted. (**F**–**T**) Soft agar colony assays to examine the effect of NP-G2-044 on the growth of various bladder cancer cells under 3D conditions. (**F**,**I**,**L**,**O**,**R**) The number of colonies of various bladder cancer cells in the absence of any drugs (control), and in the presence of NP-G2-044, cisplatin, or NP-G2-044 + cisplatin. (**G**,**J**,**M**,**P**,**S**) The average volume of individual colonies of various bladder cancer cells in the absence or presence of NP-G2-044. (**H**,**K**,**N**,**Q**,**T**) Representative images of colonies of various bladder cancer cells in the absence or presence of NP-G2-044. The data are presented as mean ± SEM. *n* = 3. **, *p* < 0.001. The scale bar, 50 μm.

In our previous studies with breast cancer cells (except for the EGFR-high triple-negative breast cancer cells), fascin inhibitors did not inhibit the growth of these tumor cells [31,32]. To investigate whether the fascin inhibitor NP-G2-044 has any effect on the growth of urinary bladder carcinoma cells, we used various experimental approaches to examine the cell growth in culture plates under 2D experimental conditions and in soft agar under 3D experimental conditions. When bladder cancer cells T24, 253J, MB49, TCCSUP, and J82 cultured in the absence and presence of a high concentration of NP-G2-044 (~10-fold higher than the IC_50_ values), no inhibitory effect on the cell growth was observed for all of these 5 bladder cancer cell lines (Figure 2A–E). As positive controls, cisplatin (50 μg/mL) and 5-FU (100 μM) inhibited the growth of these bladder cancer cells, as previously reported [37,38] (Figure 2A–E). Furthermore, the addition of NP-G2-044 did not interfere with the inhibitory effects of cisplatin and 5-FU (Figure 2A–E). These data show that NP-G2-044 did not inhibit the growth of these bladder cancer cells under 2D culture conditions.

To study the potential effect of NP-G2-044 on the growth of bladder cancer cells under a 3D experimental condition, we monitored the growth of the bladder cancer cells using the soft agar colony formation assay. These carcinoma cells were mixed with soft agar, and the number of colonies was counted after 14 days. As shown in Figure 2F,I,L,O,R, NP-G2-044 treatment did not decrease the number of colonies growing in soft agar from these bladder cancer cells. As a positive control, cisplatin decreased the number of colonies formed by these bladder cancer cells (Figure 2F,I,L,O,R). However, we noticed that NP-G2-044 decreased the volumes of individual colonies from all of these 5 bladder cancer cells (Figure 2G,H,J,K,M,N,P–T). The volume decrease ranged from 65% to 84% among these 5 bladder cancer cell lines (Figure 2G,J,M,P,S). A possible explanation is that, as we observed before, fascin inhibitor-treated cells were without filopodia and were rounded, compared to untreated cells with filopodia which were extended [31,39]. This might underlie the volume differences. Furthermore, we showed that NP-G2-044 did not induce apoptosis in these bladder cancer cells. Taken together, the above data show that although NP-G2-044 has no effect on the growth and apoptosis of bladder cancers cells, it reduces the volumes of individual colonies formed in soft agar.

### 2.3. Fascin Inhibitor Reduces Cell Adhesion

**Figure 3:**
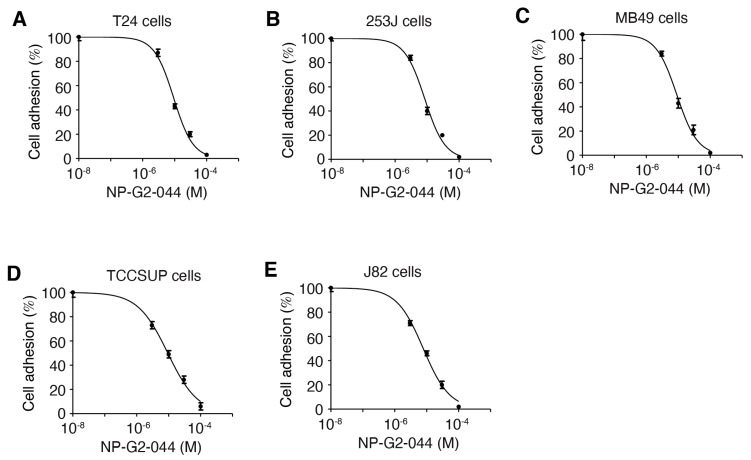
NP-G2-044 decreases the adhesion of bladder cancer cells. The effect of NP-G2-044 on cell adhesion of various bladder cancer cells was quantified. Different concentrations of NP-G2-044 were used to treat the cells. Cell adhesion was calculated using the number of adherent cells in the presence of NP-G2-044 divided by the number of adherent cells in the absence of NP-G2-044. (**A**) T24 human bladder cancer cells. (**B**) 253J human bladder cancer cells. (**C**) MB49 mouse bladder cancer cells. (**D**) TCCSUP human bladder cancer cells. (**E**) J82 human bladder cancer cells. Data were analyzed and fitted using GraphPad. Data are presented as mean ± SEM. n = 3.

Since fascin is involved in focal adhesion formation [32,40], we investigated whether NP-G2-044 affects the adhesion of bladder cancer cells. T24, 253J, MB49, TCCSUP, and J82 bladder cancer cells grew in laminin-coated plates, with or without different concentrations of NP-G2-044. After one hour, nonadherent cells and adherent cells were quantified. NP-G2-044 inhibited the adhesion of all five bladder cancer cell lines with IC_50_ values of 7.8–9.4 μM (Figure 3). Given the 99.7% plasma protein binding of NP-G2-044 (in the presence of 100% serum) [35,36], the corresponding IC_50_ values for free NP-G2-044 are 0.23–0.28 μM (in the presence of 10% of serum). These data demonstrate that NP-G2-044 inhibits the cell adhesion of bladder cancer cells.

### 2.4. Increases in the Overall Survival of Mice Bearing Bladder Cancer by Treatments with NP-G2-044 Alone and in Combination with Cisplatin

**Figure 4:**
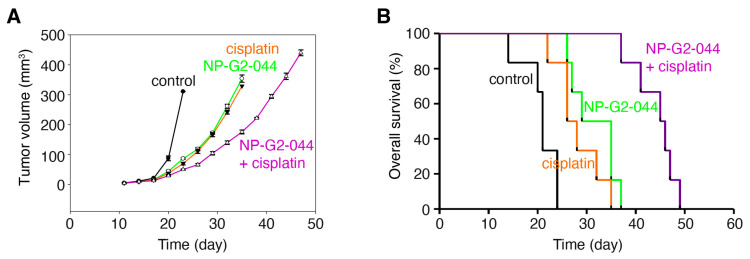
NP-G2-044 and cisplatin increase the overall survival of mice bearing bladder tumor. C57BL/6 mice implanted with MB49 mouse bladder cancer cells were treated with control solvent, NP-G2-044, cisplatin, or a combination of NP-G2-044 + cisplatin. (**A**) Primary tumor volumes were measured every 3 days. Data are shown as mean ± SEM. (**B**) Overall survival of these mice was analyzed. Death was used as the endpoint. Each group had six mice.

Currently, there are no therapeutics that specifically target metastasis for clinical use. Although NP-G2-044 did not induce apoptosis of bladder cancer cells, it is possible that its anti-migration effect, when combined with cytotoxic agents, such as cisplatin, will lead to a more robust clinical benefit. To demonstrate NP-G2-044’s effect on overall survival when combined with cisplatin, we examined the overall survival of tumor-bearing mice. C57BL/6 mice with an intact immune system were implanted with MB49 mouse bladder carcinoma cells, a syngeneic mouse model of bladder cancer widely used for 40 years [41]. The mice were divided into four treatment groups: control solvents, NP-G2-04 alone, cisplatin alone, and NP-G2-044 combined with cisplatin (Figure 4). Primary tumor growth was decreased ~72% (*p* < 0.001) by NP-G2-044 treatment when compared with the control group (using the primary tumor volumes on Day 23 for comparisons) (Figure 4A). Similarly, cisplatin decreased the tumor growth by ~78% (*p* < 0.001) (Figure 4A). A combination of NP-G2-044 and cisplatin decreased the tumor growth by ~84% (*p* < 0.001) (Figure 4A). Furthermore, the median overall survival increased ~52% by NP-G2-044 treatment when compared with the control group (log-rank test, *p* < 0.001) (Figure 4B). The median overall survival increased ~29% by cisplatin (log-rank test, *p* = 0.004) (Figure 4B). The combination of NP-G2-044 and cisplatin increased the median overall survival by ~117% (log-rank test, *p* < 0.001) (Figure 4B). These studies indicate that NP-G2-044 alone and in combination with cisplatin can extend the lives of mice bearing bladder carcinoma.

### 2.5. Increases in the Overall Survival of Mice Bearing Bladder Cancer by Treatment with NP-G2-044 in Combination with Anti-PD-1 Antibody

**Figure 5:**
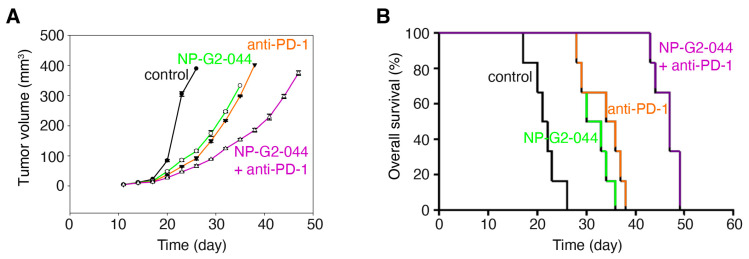
Overall survival increases of mice bearing bladder tumor by NP-G2-044 and anti-PD-1 antibody. C57BL/6 mice implanted with MB49 mouse bladder cancer cells were treated with control solvent, NP-G2-044, anti-PD-1 antibody, or a combination of NP-G2-044 + anti-PD-1 antibody. (**A**) Primary tumor volumes were measured every 3 days. Data are shown as mean ± SEM. (**B**) Overall survival of these mice was analyzed. Death was used as the endpoint. Each group had six mice.

We further investigated the effect on overall survival by fascin inhibitors in combination therapy with immune checkpoint inhibitors, such as the anti-PD-1 antibody using the above syngeneic mouse model. The tumor-bearing mice were randomized into four treatment groups: control IgG, NP-G2-044 alone, anti-PD-1 antibody alone (administered on days 11, 13, 15 and 17) [42], NP-G2-044 + anti-PD-1 antibody (Figure 5A,B). Primary tumor growth was decreased by NP-G2-044 treatment ~72% (*p* < 0.001) when compared with the control group (using the primary tumor volumes on Day 23 for comparisons) (Figure 5A). Similarly, anti-PD-1 antibody decreased the tumor growth by ~79% (*p* < 0.001) (Figure 5A). A combination of NP-G2-044 and anti-PD-1 antibody decreased the tumor growth by ~85% (*p* < 0.001) (Figure 5A). Furthermore, the overall survival of these mice was monitored (Figure 5B). The median overall survival increased ~47% by NP-G2-044 treatment (log-rank test, *p* < 0.001) (Figure 5B) when compared with the control group. The median overall survival increased ~63% by anti-PD-1 antibody (log-rank test, *p* = 0.001) (Figure 5B). The combination of NP-G2-044 and anti-PD-1 antibody increased the median overall survival by ~119% (log-rank test, *p* < 0.001) (Figure 5B). All together, these data demonstrate that NP-G2-044 can act with anti-PD-1 therapy to increase the overall survival of mice bearing bladder cancer.

## 3. Discussion

We have shown here that a new fascin inhibitor inhibits the migration of bladder carcinoma cells. This is consistent with the biochemical functions of fascin in filopodial formation and actin cytoskeletal reorganization, which are necessary for tumor cell migration. This fascin inhibitor also decreases bladder tumor cell adhesion. Focal adhesions are dynamic complexes that allow the cell to communicate with and respond to its environment. Using fluorescence recovery after photobleaching and time-lapse confocal live-cell imaging, we showed that NP-G2-044 treatment decreased the assembly and disassembly rates of focal adhesions in breast cancer cells [32]. Upon NP-G2-044 treatment, the focal adhesions were more stable. Furthermore, fascin was observed to accumulate in focal adhesions by total internal reflection fluorescence microscopy [40]. Fascin also inhibited myosine II activity and prevented the association of myosine II with F-actin filaments, implying that fasci inhibitor treatment might increase the cell contractility. Cells with knock-downed fascin generated higher tensile forces and migrated slower. Moreover, fascin was shown to modulate the remodeling of mitochondrial actin filaments to promote tumor metastasis [43]. From our previous studies, the migration sensitivity to fascin inhibitors always indicates a metastasis sensitivity to fascin inhibitors [31,32]. In our previous studies with different types of cancers, we always observe a correlation of the blocking effect on tumor cell migration in vitro and the inhibitory effect on tumor metastasis, leading to the increase in overall survival of tumor-bearing mice. Thus, fascin inhibitors will prevent bladder cancer spreading and can be used to treat metastatic bladder cancers. In addition, we showed that NP-G2-044 did not inhibit the growth of bladder cancer cells under 2D culture conditions. However, NP-G2-044 decreased the volumes of individual colonies from bladder cancer cells in soft agar colony formation assays under 3D experimental conditions. One possible explanation is that fascin inhibitor-treated cells are without filopodia, rounded, and smaller in sizes, compared to untreated cells. Furthermore, NP-G2-044 did not induce apoptosis of bladder cancers.

Given that fascin inhibitors do not induce apoptosis of bladder cancer cells, we investigated the combinatory therapy of NP-G2-044 and a chemotherapeutics agent, cisplatin, which is widely used for treating metastatic bladder cancers. We have shown that NP-G2-044 alone and cisplatin alone decreased the primary tumor growth and increased the overall survival of mice with bladder cancers. Notably, the combination of NP-G2-044 and cisplatin decreased the tumor growth and increased the median overall survival to an even greater degree. These data strongly demonstrate that NP-G2-044 together with chemotherapies should help patients with metastatic bladder cancers. These likely reflect the results of NP-G2-044’s anti-metastasis ability and the cytotoxic outcome of cisplatin. Moreover, given the recent approval of using immune checkpoint inhibitors, such as anti-PD-1 antibodies for metastatic bladder cancers, we tested the combinational use of NP-G2-044 and anti-PD-1 antibody in a syngeneic mouse model of bladder cancers. The combination treatment of NP-G2-044 + anti-PD-1 antibody led to a greater extension of median overall survival of tumor-bearing mice than the mice treated with the anti-PD-1 antibody alone. Recently, we have shown that NP-G2-044 could increase intra-tumoral dendritic cell activation, and thus, anti-cancer immunity [36]. Recently, effective anti-PD-1 treatment was shown to require intratumoral dendritic cells producing IL-12, which stimulates antitumor CD8^+^ T cell immunity [44]. Anti-PD-1 antibody activated CD8^+^ T cells release IFN-γ, which can further activate intratumoral dendritic cells. This positive feedback between intratumoral dendritic cells and T cells is essential for an effective anti-PD-1 immunotherapy [44]. Currently, only a small portion of bladder cancer patients derives clinical benefits from checkpoint immunotherapy, the addition of NP-G2-044 to the checkpoint immunotherapy should advance the care of bladder cancer patients. Our pre-clinical studies, together with the bladder cancer cells presented here, suggest possible clinical uses of fascin inhibitors as new bladder cancer treatments in combination with chemotherapies or checkpoint immunotherapies.

## 4. Materials and Methods

### 4.1. Mouse Colony

Female C57BL/6 mice (female 6~8-week-old) were purchased from Charles River Labs. Studies using mice were performed in compliance with the Institutional Animal Care and Use Committee of Weill Cornell Medical College of Cornell University (Protocol #0709-670A). All mice were housed in the facility of the Research Animal Resource Center of Weill Cornell Medical College of Cornell University.

### 4.2. Boyden-Chamber Cell Migration Assay

As described previously, bladder cancer cells (1 × 10^4^) suspended in 200 μL starvation medium were added to the upper chamber of an insert (6.5 mm diameter, 8 μm pore size; Becton Dickson), and the insert was placed in a 24-well plate containing 400 μL starvation medium with 10% FBS [31,32]. When used, fascin inhibitors were added to both the upper and the lower chambers. Migration assays were performed for 48 h and cells were fixed with 10% paraformaldehyde. Cells were stained with crystal violet, and cells on the upper side of the insert were removed with a cotton swab. Five different fields on the lower side of the insert were photographed, and the migrated cells were counted. Migration index was expressed as relative number of migrated cells in the presence of fascin inhibitors over in the absence of fascin inhibitors.

### 4.3. Cell Growth Assay

1 × 10^5^ T24, 253J, MB49, TCCSUP, or J82 cells were seeded in a 6-well plate on day 1. Control solvent, cisplatin (50 μg/mL), 5-FU (100 μM), or NP-G2-044 (100 μM), was added to the plates. On days 3, 5, and 7, cells were collected and counted from three separate wells.

### 4.4. Soft Agar Colony Formation Assay

1 × 10^3^ T24, 253J, MB49, TCCSUP, and J82 cells were suspended in 0.5 mL of 0.3% low melting point agar (Merck) in DMEM with 10% FBS and control solvent or the inhibitors (added as in the cell growth assay above). This suspension was overlaid on pre-solidified 0.6% agar in the same medium in 24-well plates, as previously described [45]. Growth medium with control solvent or the inhibitors was layered over the agar every 3 days for 14 days. The colonies were stained with 0.1% crystal violet for 1 h at room temperature and counted under an inverted microscope. The colony volume was calculated according to V = (4/3) πr^3^.

### 4.5. Caspase Activity Assay

Cells (T24, 253J, MB49, TCCSUP, and J82) were treated with NP-G2-044 at different concentrations (0, 3, 10, 30, 100 μM). Floating cells and attached cells (collected by trypsinization) were lysed. Twenty micrograms of cell lysates were then mixed with caspase-3 (Apopain) substrate Rhodamine 110 (AnaSpec; final concentration 1.5 μM) on ice in a final volume of 20 μL. Samples were then immediately loaded on 96-well plates and fluorescence was measured at 30 °C for 180 min using TECAN at excitation and emission 485 and 535 nm, respectively. Average relative fluorescence units at 120 min were plotted, and data are shown as mean ± SEM of at least three independent assays.

### 4.6. Cell Adhesion Assay

96-well-plates were coated with Laminin-1 (10 μg/mL, 70 μL/well) at 4 °C overnight. The pates were washed with PBS once, blocked with 1% BSA (in DMEM) at 37 °C in CO_2_ incubator for 1 h, and washed with PBS. 5000 cells were added to each well. Different concentrations of NP-G2-044 were included. After incubating in a CO_2_ incubator at 37 °C for 1 h, nonadherent cells were washed away with PBS. The plates were fixed with 4% paraformaldehyde for 30 min at RT. After washing with PBS, the plates were stained with 0.1% Crystal Violet for 30 min at RT and washed with PBS. After the plates were dry, the number of adherent cells was counted using an inverted microscope.

### 4.7. Overall Survival Analysis in Mice

For the studies of NP-G2-044 and cisplatin, 6–8-week-old female C57BL/6 mice were randomly divided into four equal groups (control, NP-G2-044, cisplatin, and NP-G2-044 + cisplatin). MB49 cells were harvested to prepare cell suspensions containing 1 × 10^6^ cells/mL which were injected into the right flank subcutaneous tissues at 100 µL/mouse. On day eight, when the maximum diameter of the tumors reached 2 mm, NP-G2-044 (100 mg/kg) was given by oral gavage daily. On day 11, cisplatin (3 mg/kg, once weekly) was intraperitoneally injected. The primary tumor volume was measured using calipers every 3 days and calculated with the following formula: V (mm^3^) = (Length × Width^2^)/2. Mice were observed daily for mortality.

For the studies with NP-G2-044 and anti-PD-1 antibodies, MB49 cells (1 × 10^6^) suspended in PBS-matrigel (*v/v*, 1:1) were subcutaneously injected into the right flank of female C57BL/6 (6–8-week-old) mice on day one. The mice were treated with control solvent, NP-G2-044, anti-PD-1 antibody, or NP-G2-044 + anti-PD-1 antibody. Starting on day eight, NP-G2-044 (100 mg/kg) was given once a day by oral gavage. On days 11, 13, 15, and 17, anti-PD-1 antibody was given by i.p. at 10 mg/kg per mouse. Control solvent was given to control group according to their body weight. The primary tumor growth and death of mice were recorded.

### 4.8. Statistical Analysis

Median overall survival analysis was performed by log-rank test with significance defined as *p* < 0.05. For other studies, the statistical significance of differences between groups was evaluated by the Holm multiple comparisons test (Prism 9, GraphPad). Results were considered significant at *p* < 0.05. Data are representative of at least three independent experiments.

## 5. Conclusions

Fascin inhibitors, alone or in combination with chemotherapy or immuno-oncology therapy, can be used as new treatments for bladder cancer. 

## Data Availability

The data presented in this study are available in this article.
